# Subtypes of high-grade breast ductal carcinoma in situ (DCIS): incidence and potential clinical impact

**DOI:** 10.1007/s10549-023-07016-9

**Published:** 2023-07-15

**Authors:** Hossein Schandiz, Daehoon Park, Yan Liu Kaiser, Marianne Lyngra, Inger Solvang Talleraas, Jürgen Geisler, Torill Sauer

**Affiliations:** 1 Department of Pathology, Akershus University Hospital, Lørenskog, Norway; 2 Department of Oncology, Akershus University Hospital, Lørenskog, Norway; 3 Department of Clinical Molecular Biology (EpiGen), Akershus University Hospital (AHUS), Lørenskog, Norway; 4 Department of Pathology, Oslo University Hospital, Oslo, Norway; 5 Institute of Clinical Medicine, Faculty of Medicine, University of Oslo, Campus AHUS, Oslo, Norway

**Keywords:** Ductal carcinoma in situ, Invasive breast carcinoma, Immunohistochemistry, Molecular subtypes, Heterogeneity

## Abstract

**Objective:**

The purpose of this study was to investigate and classify the molecular subtypes of high-grade ductal carcinoma in situ (DCIS) and identify possible high-risk subtypes. The heterogenicity of DCIS with variable clinical and histopathological presentations has been recognized. Nevertheless, only histopathological grading and diameter are currently implemented in clinical decision-making following the diagnosis of DCIS. The molecular subtypes of DCIS and their IHC surrogate markers have not been defined in conventional treatment guidelines and recommendations. We applied the definitions of molecular subtypes according to the IHC surrogate markers defined for IBC and subclassified high-grade DCIS, accordingly.

**Methods:**

Histopathological specimens were collected, revised, and regraded from 494 patients diagnosed with DCIS between 1996 and 2018. Other in situ and papillary lesions observed in breast biopsies were excluded from this study. 357 high-grade DCIS cases were submitted to IHC analysis. The markers investigated were ER, PR, HER2, and Ki67.

**Results:**

45 cases were classified as grade 1, 19 as grade 2, and 430 as grade 3. Sixty patients with high-grade DCIS had an additional invasive component in the surgical specimen. Thirty-three patients were diagnosed with recurrent DCIS or invasive cancer (minimum one year after their primary DCIS diagnosis). The proportions of luminal A and luminal B HER2-negative subtypes varied depending on whether 2011 or 2013 St. Gallen Consensus Conference guidelines were adopted. Luminal A was the most prevalent subtype, according to both classifications. The luminal B HER2-positive subtype was found in 22.1% of cases, HER2-enriched subtype in 21.8%, and TPN subtype in 5.6%. There were strong indications that HER2-enriched subtype was significantly more frequent among DCIS with invasive component (*p* = 0.0169).

**Conclusions:**

High-grade DCIS exhibits all the molecular subtypes previously identified in IBC, but with a somewhat different distribution in our cohort. HER2-enriched subtype is substantially related to the presence of an invasive component in DCIS; consequently, it is regarded as a high-risk entity.

**Supplementary Information:**

The online version contains supplementary material available at 10.1007/s10549-023-07016-9.

## Introduction

Ductal carcinoma in situ (DCIS) of the breast is a heterogeneous disease with a considerable variation in clinical presentations as well as long-term prognosis [1]. Similarly, DCIS has various histopathological presentations. In general, DCIS is defined as a neoplastic proliferation of epithelial cells in the ducts and glands of the mammary gland that do not penetrate the myoepithelium-basement membrane barrier [2]. It is unclear why some DCIS may reach 7–8 cm in size and remain in situ, whereas others become invasive carcinomas at an early stage [3]. Some studies have indicated that poorly differentiated DCIS may gradually evolve from well-differentiated DCIS through genetic defects amplified by p53 mutations [4, 5]. The cellular or nuclear atypia of DCIS varies from discrete to gross and pronounced. The risk of progression to invasive lesions varies according to atypia [6]. DCIS is still mainly treated according to the grade and extent of the lesion, and probably many of these lesions are currently overtreated [7]. The variation in demographic and geographic factors may have an impact in undertreatment of patients [8]. Quantifying the long-term benefits of treating asymptomatic DCIS that may or may not progress to invasive breast carcinoma (IBC) remains challenging. However, there is evidence that only a subfraction of DCIS develops into IBC [9]. A substantial molecular genetic analysis has established a comprehensive molecular subclassification with strong implications for the treatment of IBC which is still lacking for DCIS [10, 11]. In the routine diagnosis of IBC, surrogate markers are used by immunohistochemistry (IHC), such as estrogen receptor (ER), progesterone receptor (PR), and human epidermal growth factor receptor 2 (HER2), and  the Ki67 proliferation index, as tools to estimate the risk of micrometastasis and relapse, as well as to stratify for treatment [12]. Individual results for these markers have resulted in the development of complex treatment algorithms [13]. In contrast, all DCIS grade 3 patients are still given the same treatment, according to grade and extent, and generally do not have any additional prognostic markers [14]. Radiological microcalcifications are frequently detected [15, 16], and a large proportion of these are histologically in situ lesions (DCIS). Thus, DCIS has become a relatively common diagnosis after the introduction of screening mammography, representing up to 20 – 25% of all incident breast malignancies in industrialized countries [17]. The mammography program in Norway started in 1996, and DCIS represents approximately 20% of diagnosed premalignant and malignant incidents in the breast [18, 19]. A few studies have shown that the molecular subtypes of IBC are also present in DCIS, albeit not with the same pattern of distribution [20, 21]. Strand et al. [22] have published a molecular genetic classification based on 774 DCIS cases. IHC was not performed; thus, the correlation to the IHC surrogate markers is unknown. In line with the few studies conducted on DCIS thus far, heterogeneity of molecular subtypes has largely been found in high-grade lesions [23, 24]. Thus, our study aimed to investigate the distribution of the molecular subtypes of DCIS grade 3.

## Materials and methods

### Material

Our study included histopathological specimens from 494 patients diagnosed with DCIS of the breast between 1996 and 2018. Cases with a primary biopsy SNOMED code of M85002 (the diagnostic code for DCIS) were searched in the database (Doculive Patologi) of the Department of Pathology, Akershus University Hospital, Norway. We chose this code to ensure that the vast majority of biopsies had been performed because of the detection of microcalcifications on mammography that were suspicious for DCIS and not a mammographic tumor. Other in situ and papillary lesions observed in breast biopsies were excluded. We included microinvasive and small invasive lesions up to 10 mm (pT1b) [25] if the surgical specimen contained these findings. All cases with larger lesions were excluded to ensure that the indication for biopsy was microcalcification and not a radiological tumor with concurrent microcalcifications. All living patients received an information letter in which the purpose of the project was described. The text of the information letter was approved by the Regional Committee for Medical and Health Research Ethics (REK). A prepaid returning envelope was enclosed in the letters patients received, in addition to a sheet to sign and return if they objected. If they agree, they would not need to undertake any actions. We received reservations from ten patients (eight were as grade 3, one was as grade 2 and one was as grade 1); as a consequence, their cases were excluded from further examinations. The remaining 484 cases were subjected to our analysis. Materials from deceased patients could be used without any consent or information from their relatives. We are aware of the challenges regarding the grading of DCIS [7], mainly due to the different classification systems and interobserver evaluations between pathologists. Hence, two experienced breast pathologists revised and regraded all cases. If they disagreed, a third experienced breast pathologist evaluated each case. The final grade was set as simple majority. The extent of the DCIS was recorded when this information was available. In the case of multifocal lesions, separate foci were added and the total extent was noted. Any DCIS or invasive tumor in the ipsilateral or contralateral breast diagnosed at least one year after the primary DCIS diagnosis, as well as metastases and non-breast tumors discovered in the department database, were recorded.

### Methods

**Fig. 1:**
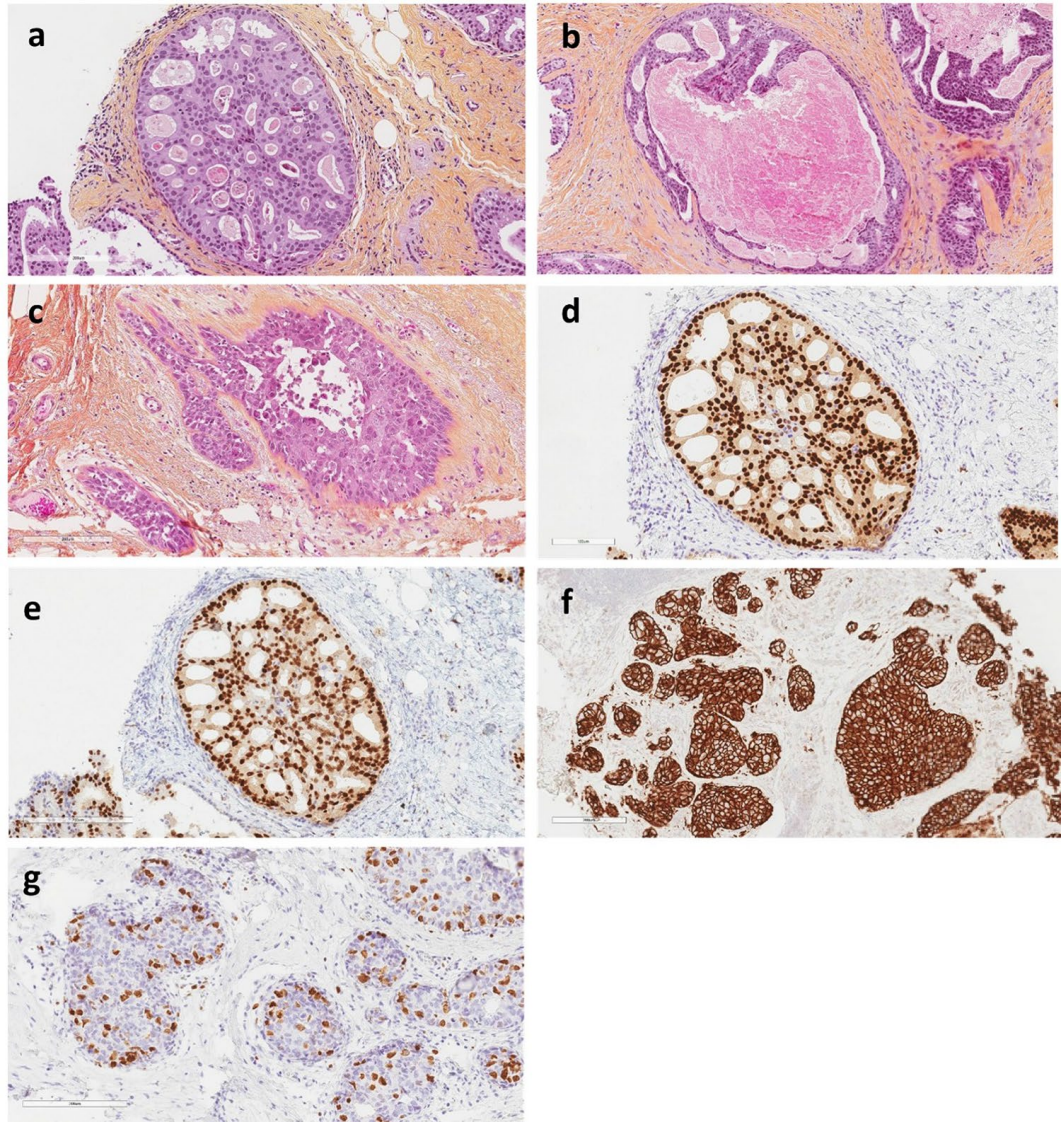
**a** Hematoxylin phloxine saffron (HPS) stain of DCIS grade 1, with small and monotonous epithelial nuclei. 20X magnification. **b** HPS stain of DCIS grade 2, small and monotonous epithelial nuclei with comedo-type necrosis. 20X magnification. **c** HPS staining of DCIS grade 3 enlarged and pleomorphic epithelial nuclei. 20X magnification. **d** IHC staining of DCIS showing strong nuclear estrogen receptor (ER) expression. 20X magnification. **e** IHC stain of DCIS showing strong nuclear progesterone receptor (PR) expression. 20X magnification. **f** IHC staining of DCIS showing strong and complete membranous expression (3+) of human epidermal growth factor receptor 2 (HER2). 20X magnification. **g** IHC staining of DCIS showing nuclear Ki67 expression. 20X magnification

In Norway, DCIS is graded using the Van Nuys classification [26] (Supplementary Table 1). It does not take into account growth patterns but is based on the nuclear size, which is compared to the size of RBC (≶ 2X) and the presence or absence of comedo-type necrosis (Fig. 1a–c). Given the nuclear size, this is essentially a two-tier classification. In contrast to most other grading systems for DCIS, which are three-tiered systems. The practical result was that a large majority of DCIS cases in our study were diagnosed as high-grade. Thus, this group was selected for the present study. Contrary to other studies [23], we excluded all other in situ lesions, which account for approximately 20% of the precancerous lesions in the mammary gland [27, 28]. If we had included these, the proportion of high-grade DCIS in our study would have been 72%. The molecular subtypes of DCIS and their IHC surrogate markers have not been defined in any guidelines or treatment recommendations. Thus, we implemented definitions and classifications of molecular subtypes according to the IHC surrogate markers defined for IBC at St. Gallen International Expert Consensus Conferences in 2011 and 2013 (Supplementary Table 2).

#### Immunohistochemistry

IHC analysis of 357 DCIS grade 3 cases stained for ER, PR, HER2, and Ki67 were subjected to our further study. ER and PR receptor positivity was defined as ≥ 1% positive tumor cells, according to the updated guidelines of the American Society of Clinical Oncology (ASCO) and the College of American Pathologists (CAP). Ki67 IHC was evaluated by counting 200 DCIS cells (negative and positive intraductal epithelial cells) in two separate hotspot foci and the ratio was calculated. The cut-off values were reported to be 14% and 20% respectively, in compliance with recommendations from St. Gallen consensus meetings in 2011 and 2013 [29–31]. HER2 IHC was scored based on ASCO/CAP guidelines, as in routine diagnostic procedures [32]. HER2 silver in situ hybridization (SISH) was performed when IHC was 2 + [33]. IHC staining for ER, PR, HER2, and Ki67 was performed using a Dako Autostainer (Agilent). HER2 SISH was performed on a Roche Diagnostics BenchMark system using a fully automated Ultra-IHC/ISH staining module [34]. CC2 was used as the buffer. The details of the antibodies and HER2 SISH probes are described in Supplementary Table 3. The IHC protocol for the Dako Autostainer is shown in Supplementary Table 4. A positive control tissue was included in each run. Subtypes are described separately, according to 2011 and 2013 St. Gallen International Expert Consensus Conferences.

##### Statistical analysis

GraphPad Prism version 9.4 was chosen for the statistical calculations. To calculate the statistical significance, we executed different tests. The Mann–Whitney U test, a non-parametric test, was performed for independent measurements when variables between two independent groups were compared. The Kruskal–Wallis test, a non-parametric test, was chosen to compare independent measurements for variables between multiple subtypes. One-way ANOVA and parametric analysis were selected when performing normally distributed multiple comparisons and if the Shapiro–Wilk normality test was passed. Fisher's exact test was performed to calculate the *p*-values when comparing two proportions using a contingency table. Statistical significance was set a priori at *p* < 0.05.

## Results

**Fig. 2:**
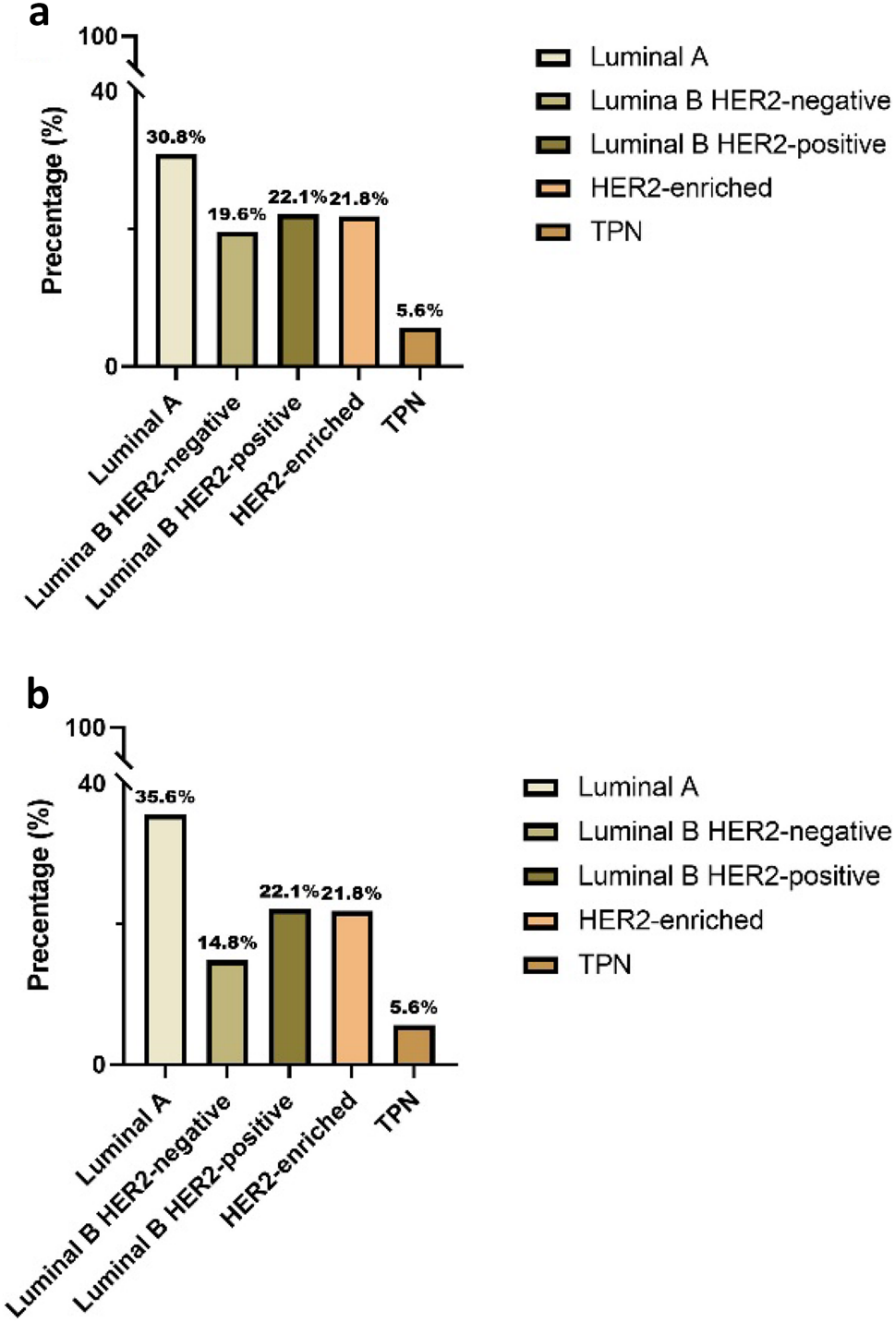
**a** Distribution of molecular subtypes, calculated and classified according to the 2011 St. Gallen recommendations. **b** Distribution of molecular subtypes, calculated and classified according to 2013 St. Gallen recommendations

Details of the histopathological findings and grading are shown in Table 1 and include both “pure” DCIS (without an invasive component) and DCIS with an additional invasive component. We received reservations from 10 patients who were excluded from further analysis. Of the  remaining 484 patients, 44 (9.0%) were grade 1, 18 (4.0%) were grade 2, and 422 (87%) were grade 3. We excluded papillary and in situ lesions of the breast. The percentage of high-grade DCIS in our study would have been 72% if we had included these. A primary discrepancy in regrading was found in 41 cases; in 58% of these cases, the final grade was DCIS grade 3 (n  = 24). A report of an in situ or invasive carcinoma in the same or contralateral breast diagnosed at least one year after the primary diagnosis and treatment was found in 33 cases (6.8%). An overview of the later events is presented in Table 2.  According to our local database 70 patients were registered as deceased. Only one patient was known to have passed away from metastatic IBC. We did not have access to the cause of death registry of deceased patients. The age of the patients ranged from 33 to 90 years (mean 57, and median 57). The DCIS extent ranged from 0.5 to 150 mm, (mean 28.4, and median 20). Details regarding the distribution of the extent of DCIS grade 3 and the age of the patients for each subtype are shown in Supplementary Tables 5a and 5b. Samples from 422 DCIS grade 3 were intended for IHC analysis. However, 53 cases, including 13 with an invasive component, were lost due to missing formalin-fixed paraffin-embedded (FFPE) samples in the department archive, and 12 samples lost their tissues during the IHC steps. Subsequently, 357 DCIS grade 3 cases, with all IHC markers for subtyping, were identified (Fig. 1d–g). The distribution of molecular subtypes according to the IHC surrogate markers are displayed in Tables 3 and 4 and Fig. 2a and b. Briefly, 64 patients were found to have an additional invasive carcinoma component (pT1b or less) in the surgical specimen from the same breast. 21 had an extent of ≤ 1 mm (pTmic), 27 had an extent of > 1 mm but ≤ 5 mm, (pT1a) and  16 had an extent of > 5 mm but ≤  10 mm (pT1b). Four cases were low-grade and the remaining 60 were high-grade. Further investigations of the invasive components were not included in this study. Tables 5 and 6 demonstrate the distribution of molecular subtypes based on IHC surrogate markers for 51 cases with invasive components.

**Table 1:** Histopathological findings and grading in 484 cases of DCIS

	Grade	Number	%
Grading		494	
		10 reservations were excluded	
	G1	1	10
	G2	1	10
	G3	8	80
		484 cases remained for further assessments	
	G1	44	9.0
	G2	18	4.0
	G3	422	87
Discrepancy cases; final grade (n = 41/484)		41	8.5
	G1	11	27
	G2	6	15
	G3	24	58
All cases with invasive components (n = 64/357)	G1 (n = 3)	n = 1 (pTmic), n = 2 (pT1a)	4.5
	G2 (n = 1)	n = 1 (pT1a)	1.5
G3 cases with invasive components (n = 60/64)	G3 (n = 60)	60	94
		20 (pTmic)	33
		24 (pT1a)	40
		16 (pT1b)	27
G3 cases with IHC (n = 51/357)		16 (pTmic)	31
		20 (pT1a)	39
		15 (pT1b)	30

Extent (mm)	Min	Max	Median	Mean
All cases (n = 484)	0.5	150	20	28.4
All cases with invasive component (n = 64)	4	120	30	37
G1/G2 (n = 4)	9	80	25	35

**Table 2:** Later events occurring after primary DCIS diagnosis and treatment

	Dead (= n)	Number (n = 484)	%	Years after primary DCIS diagnosis and treatment
All subsequent DCIS or IBC	6	33	6.8	> 1 year
Primary was G1		5		1–5
Primary was G2		2		5–7
Primary was G3		26		> 1 year
Axillary metastases	0	3		> 1 year
Locoregional recurrence and liver metastasis	0	1		3 (locoregional)
				4 (liver metastasis)
Dead of metastatic IBC^#^	1	1		3 (liver metastasis)
				4 (pleura metastasis)
Angiosarcoma of the breast	1	1		8
Small cell lung carcinoma	1	1		5
Lung adenocarcinoma	1	1		10
Malignant thymoma	1	1		9
Thymic T-cell lymphoma	1	1		6
Metastatic* carcinoma (in pericardial effusion) of unknown origin	1	1		2 months

^#^The patient did not have an autopsy, but several metastatic effusion specimens were registered in the pathology database immediately before death

*DCIS was an “incidental” finding while searching for a primary lesion

**Table 3:** The distribution of molecular subtypes among “pure” DCIS and DCIS with an invasive component, according to the IHC surrogate markers, calculated and classified in the manner of 2011 St. Gallen recommendations

Subtypes	Luminal A	Luminal B HER2-negative	Luminal B HER2-positive	HER2-enriched	TPN	
n = 357 (100%)	n = 110	n = 70	n = 79	n = 78	n = 20	
Percentage of all	30.8	19.6	22.1	21.8	5.6	100
Percentage of “pure” DCIS	86	90	89	77	90	86
Percentage of DCIS w/invasion	14	10	11	23	10	14

**Table 4:** The distribution of molecular subtypes among “pure” DCIS and DCIS with an invasive component, according to the IHC surrogate markers, calculated and classified in the manner of 2013 St. Gallen recommendations

Subtypes	Luminal A	Luminal B HER2-negative	Luminal B HER2-positive	HER2-enriched	TPN	
n = 357 (100%)	n = 127	n = 53	n = 79	n = 78	n = 20	
Percentage of all	35.6	14.8	22.1	21.8	5.6	100
Percentage of “pure” DCIS	87	90	89	77	90	86
Percentage of DCIS w/invasion	13	10	11	23	10	14

**Table 5:** The molecular subtypes of 51 cases with an invasive carcinoma component, according the IHC surrogate markers, calculated and classified in the manner of 2011 St. Gallen recommendations

Subtypes		Luminal A	Luminal B HER2-negative	Luminal B HER2-positive	HER2-enriched	TPN
Sum of all n = 51		n = 15	n = 7	n = 9	n = 18	n = 2
Percentage of all		29%	14%	18%	35%	4.0%
	pT					
	pTmic	5 (33%)		3 (33%)	7 (44%)	1 (50%)
	pT1a	2 (13%)	3 (43%)	4 (45%)	10 (44%)	1 (50%)
	pT1b	8 (54%)	4 (57%)	2 (22%)	1 (12%)

**Table 6:** The molecular subtypes of 51 cases with an invasive carcinoma component, according the IHC surrogate markers, calculated and classified in the manner of 2013 St. Gallen recommendations

Subtypes		Luminal A	Luminal B HER2-negative	Luminal B HER2-positive	HER2-enriched	TPN
Sum of all n = 51		n = 17	n = 5	n = 9	n = 18	n = 2
Percentage of all		33%	10%	18%	35%	4.0%
	pT					
	pTmic	5 (29%)		3 (33%)	7 (44%)	1 (50%)
	pT1a	3 (18%)	2 (40%)	4 (45%)	10 (44%)	1 (50%)
	pT1b	9 (53%)	3 (60%)	2 (22%)	1 (12%)	

The 2011 St. Gallen conference defines (Supplementary Flowchart 1a) the subtypes as follows: luminal A is defined when ER and/or PR receptors are positive, HER2 is negative, and Ki67 < 14%. When ER and/or PR receptors are positive, HER2 is negative, and Ki67 ≥ 14%, the luminal B HER2-negative subtype is defined. Luminal B HER2 positive subtype is defined when ER, PR receptors and HER2 are positive and Ki67 is at any value. The HER2-enriched subtype is defined as having ER and PR receptors that are negative, HER2 that is positive, and Ki67 of any value. Finally, triple-negative (TPN) is defined when ER and PR receptors and HER2 are negative, and Ki67 of any value.

Following the 2013 St. Gallen conference, the subtypes are defined (Supplementary Flowchart 1b) as follows: luminal A is defined when ER receptors are positive along with positive PR expression ≥ 20%, HER2 is negative, and Ki67 < 20%. Luminal B HER2-negative is defined as ER receptors that are positive and PR expression < 20%, HER2 is negative and Ki67 expression ≥ 20%, or when ER receptors are positive and Ki67 ≥ 20 or PR expression < 20%, and HER2 is negative. Luminal B HER2 positive is defined when ER and/or PR receptors are present, as well as HER2 positivity and Ki67 at any value. HER2-enriched is defined as having ER and PR negativity, HER2 positivity, and any Ki67 value. TPN is defined when ER and PR receptors and HER2 are negative, as well as Ki67 at any value.

Depending on the implementation of recommendations from St. Gallen 2011 versus 2013 meetings, we confirmed that the distribution of luminal A and luminal B HER2-negative subtypes varied. According to 2013 St. Gallen classification the proportion of luminal A was 35.6% of cases, while it was 30.8% when following the 2011 St. Gallen guidelines. By adopting the 2011 St. Gallen recommendations, we identified that luminal B cases which made up 41.7% of the total, was constituted of 19.6% HER2-negative and 22.1% HER2-positive.

Of all cases, 27.4% belonged to the non-luminal subtypes, of which 5.6% were TPN and 21.8% were HER2-enriched. Using criteria and recommendations from 2013 St. Gallen Consensus Conference revealed that the ratios of luminal A and luminal B cases were the same, 35.6% versus 36.9%; 14.8% of those with luminal B were HER2-negative and 22.1% were HER2-positive. With the TPN subtype present in 5.6% of cases and HER2-enriched subtype in 21.8% of cases, the proportion of non-luminal cases remained unchanged.

## Discussion

Our IHC results support the heterogeneity of DCIS grade 3 by demonstrating that all major molecular subtypes, as recognized for IBC, are present in DCIS grade 3. IBCs are subject to the St. Gallen classifications, yet we chose to use these to study the distributions of each individual subtype when we compared the two classifications side-by-side. These recommendations have also been described in other DCIS studies [35]. To distinguish patients with luminal A cancer from those with luminal B cancer, the 2013 St. Gallen consensus meeting introduced a 20% cut-off value for PR receptors together with an enhanced threshold for Ki67 (20%). In some studies, poor PR expression was found to be an independent prognostic and predictive factor for IBC [36]. Contrary to what ER receptors suggest, there are no promising or effective drugs that specifically target PR receptors [37, 38]. When we applied only an elevated cut-off value of 20% for PR receptors, as decided at the 2013 St. Gallen conference, the number of luminal A cases declined and the number of luminal B HER2-negative cases increased. Increasing the Ki67 threshold to 20% had an opposite effect with an elevated number of luminal B HER2-negative cases and a reduction in luminal A cases; however, when we combined these two setpoints; PR receptors at 20% and/or Ki67 at 20%, as defined by 2013 St. Gallen meeting, we observed a net gain of 17 cases in favor of luminal A subtype. When luminal A cases were added to luminal B HER2-negative cases, the total number was 180 of 357 (50.4%), regardless of St. Gallen classifications (110 + 70, St. Gallen 2011 and 127 + 53, St. Gallen 2013). The so-called "non-Ki67% sensitive" subtypes, namely luminal B HER2-positive (22.1%), HER2-enriched (21.8%), and TPN (5.6%), were unaffected. Irrespective of the Ki67 cut-off of 14% or 20%, luminal B HER2-positive and HER2-enriched subtypes constituted 157 of the 357 (44% of the total), and 27 (17.2%) of these had an additional invasive component (Tables 5 and 6).

Ki67 is considered an important prognostic proliferation marker among the luminal subtypes of IBC [39, 40]. However, agreeing on a definite cut-off for this marker has been challenging [41, 42]. There are several reasons for this, such as preanalytical and analytical assessments, interlaboratory discordance in tumor regions selected for evaluation, counting methods, and subjective assessment of staining positivity [43]. In addition, Ki67 displays a continuous distribution [44].

Our results are similar to those of a study performed by Maisonneuve et al. [45], in which the distributions of luminal A and luminal B HER2-negative cases among 9,415 patients who underwent surgery for endocrine-responsive HER2-negative breast cancer, were investigated. The relative proportions of luminal A and luminal B HER2-negative cases varied, with a net gain in favor of luminal A, when elevated setpoints for Ki67 and PR receptors were conducted. This was despite the fact that some cases had to be redefined as luminal B HER2-negative owing to a higher setpoint of the PR threshold (20%).

According to our findings, HER2-positive expression (IHC 3 + or SISH amplification) was observed in 157 samples. Of these, 79 of the 357 were luminal B HER2-positive, and 78 of the 357 were HER2-enriched, constituting 44.0% of the material. This is consistent with the findings of SA Lari et al. [46], where the mean expression rate of HER2 positivity was 40% (range, 9 – 67%). It is well known that DCIS has a higher positive expression rate of HER2 than IBC, which is approximately 15 – 20% [47–49]. In contrast, the frequency of TPN subtype is higher in IBC than in DCIS (approximately 10–20%), depending on ethnic background, underlined by the fact that this phenotype is more common in African-American patients than in Caucasian patients [50]. Perez et al. [51] reported 202 cases of high-grade DCIS, either “pure” or associated with IBC. They found the following distribution of subtypes among the “pure” DCIS: luminal A at 57.1%, luminal B at 11.9%, HER2-enriched at 16.7%, basal-like at 0%, and "not classified" (14.3%). They found no significant difference in immunophenotype frequencies between “pure” DCIS and DCIS associated with IBC (*p* > *0.05*). The differences between their findings and those of our study can be explained by the role of Ki67 in defining the subtypes. Compared to our findings and as defined at the St. Gallen meeting in 2011 (Table 3), we found that the proportions of the subtypes differed between those classified as “pure” DCIS and those classified as having an additional invasive component, in which the HER2-enriched subtype was the largest among those with an invasive component (*p*-value = 0.0169, Fisher's exact test). Thus, the HER2 subtype had the highest risk of harboring an invasive lesion in addition to the in situ component. Thorat et al. [52] reported HER2 expression (IHC 3+) in 34.4% of DCIS cases, however they did not distinguish between cases of luminal B HER2-positive and those of “pure” HER2-enriched subtype. They did not find a significant risk of a simultaneous invasive component; however, when we combined our “pure” HER2-enriched and luminal B HER2-positive cases, we found the same result. Thorat et al. found a significantly higher risk of recurrent DCIS but not invasive lesions in this group. Thus, according to our results, a significant risk of a simultaneous invasive component is linked to the HER2-enriched subtype. Furthermore, in our study the extents of HER2-enriched and luminal B HER2-positive lesions were significantly greater than that of luminal A lesional extent (adjusted *p* values < 0.0001 and 0.0063, Kruskal–Wallis test). We also found that the relative proportions of luminal A cases were identical between “pure” DCIS and DCIS with an invasive component, when the St. Gallen 2011 recommendations were conducted; 30.8% and 29.5% respectively (Tables 3 and 4). In contrast, the proportions of the remaining subtypes (luminal B HER2-negative, luminal B HER2-positive, and TPN) showed a reduction.

### Strengths of the study

A substantial number of DCIS samples (n = 494) from the surgical specimens and biopsies used to make the initial diagnoses were collected over a 22-year period. Qualified mammary pathologists assessed and rated all samples. Nationally standardized methods were used, and all IHC analyses were performed in a single laboratory.

### Limitations of the study

The analysis of IHC samples of Ki67 is a well-known challenge, causing variations in the analysis and assessment guidelines, both within a single pathology department and among different institutions. We did not have access to clinical follow-up and cause of death registry which was a limitation of our study [53].

## Conclusions

All the four main molecular subtypes recognized for IBC are also present in high-grade DCIS, resulting in a considerably heterogeneous group. Our findings suggest that the choice of the classification system can have a notable impact on the distribution of DCIS subtypes, which may have an impact on treatment options and patient outcomes. Further studies are required to identify the most precise and therapeutically beneficial system to identify high-grade DCIS subtypes. In contrast to the other subtypes, the luminal B subtypes displayed a distinguishable heterogeneity for all four surrogate IHC markers. Hence, it is crucial to devote greater focus to conducting separate studies on luminal B subtypes. Our study may contribute to a better selection of patients with high-grade DCIS in future clinical trials. There are strong indications that the HER2-enriched subtype is considered a high-risk entity among DCIS grade 3 cases, as it is significantly associated with the presence of an invasive component in our cohort (*p* value = 0.0169).

## Supplementary Information

Below is the link to the electronic supplementary material.

Supplementary file1 (DOCX 154 KB)

## Data Availability

The data contain sensitive patient information and is restricted by the local rules to not be distributed externally.

## Abbreviations

DCIS: Ductal carcinoma in situ
IBC: Invasive breast carcinoma
IHC: Immunohistochemistry
ER: Estrogen receptor
PR: Progesterone receptor
HER2: Human epidermal growth factor receptor 2
Ki67: Proliferation index
SISH: Silver in situ hybridization
FFPE: Formalin-fixed paraffin-embedded
RBC: Red blood cell
