# Supplementary material for: Subtypes of high-grade breast ductal carcinoma in situ (DCIS): incidence and potential clinical impact
Source: Breast Cancer Res Treat. 2023 Jul 15;201(2):329–38. doi: 10.1007/s10549-023-07016-9 (PMC10361903; doi:10.1007/s10549-023-07016-9)

| **Supplementary Table 1** Van Nuys classification of breast DCIS (54) | |
| --- | --- |
| DCIS grade 1 | Nuclear size < 2X RBC and without comedo-type necrosis |
| DCIS grade 2 | Nuclear size < 2X RBC and with focal comedo-type necrosis |
| DCIS grade 3 | Nuclear size ≥ 2X RBC with- or without comedo-type necrosis |

| **Supplementary Table 2** Definition of molecular subtypes for IBC, according to surrogate IHC markers (10, 11) | | | | | | | |  |
| --- | --- | --- | --- | --- | --- | --- | --- | --- |
| Luminal A | | | Hormone receptors (ER and/or PR) positive, HER2-negative, a low level of proliferation according to the Ki67 proliferation index. Luminal A tumors are low-grade, tend to grow slowly, and have the best prognosis. | | | | |  |
| Luminal B  HER2-negative | | | Hormone receptors (ER and/or PR) positive, HER2-negative, and with higher levels of Ki67 proliferation index, than luminal A tumors. | | | | |  |
| Luminal B  HER2-positive | | | Hormone receptors (ER and/or PR) positive, HER2-positive, and any value level of Ki67 proliferation index. | | | | |  |
| HER2-enriched | | | Hormone receptors (ER and PR) negative, HER2-positive, and any value level of Ki67 proliferation index. | | | | |  |
| TPN | | | Hormone receptors (ER and PR) and HER2-negative, any value level of Ki67 proliferation index. | | | | |  |
| **Supplementary Table 3** Details of antibodies and HER2 SISH probe (34) | | | | | | | |  |
| Antibody and HER2 probe | | Clone | | Reference Id. | Vendor | Dilution | Incubation time |  |
| Anti-Ki67 | | MIB-1 | | M724001-2 | Agilent | 1:200 | 20 min. |  |
| Anti-Human estrogen receptor α | | EP1 | | M364301-2 | Agilent | 1:50 | 30 min. |  |
| Anti-Human progesterone receptor | | PR 636 | | M356901-2 | Agilent | 1:100 | 30 min. |  |
| Anti-HER2/ErbB2 | | D8F12 | | #4290 | Cell Signaling | 1:200 | 60 min. |  |
| HER2 SISH | | Ventana HER2 Dual ISH DNA probe cocktail | | 8314373001 | Ventana (Roche) | Ready to use | 60 min. |  |
| **Supplementary Table 4** IHC procedure Dako Autostainer | | | | | | | |  |
| Step 1 | | Antigen retrieval was achieved in a PT-Link station by immersion into EnVision™ FLEX Target Retrieval Solution high pH (K8004, Agilent) by the heating program at 97°C for 20 minutes | | | | | | |
| Step 2 | | Endogenous peroxidase activity was quenched by incubating the slides in EnVision™ FLEX peroxidase blocking reagent (K8000, Agilent) for 5 minutes | | | | | | |
| Step 3 | | For HER2 IHC, in addition, non-specific staining was inhibited by Animal-Free blocking Solution 1x (#15019) for 30 minutes | | | | | | |
| Step 4 | | Primary antibodies Ki67 (1:200), ER (1:50), and PR (1:100) were diluted in EnVision™ FLEX Antibody Diluent (K8006, Agilent); antibody HER2 (1:200) was diluted in SignalStain® Antibody Diluent (#8112, Cell Signaling); and slides were incubated with primary antibodies for 20-60 minutes (table 3) at room temperature | | | | | | |
| Step 5 | | For ER and PR IHC, rabbit (K800921-2, Agilent) and mouse Linker (K800221-2, Agilent) were added respectively for 15 minutes for signal amplification after primary antibody incubation | | | | | | |
| Step 6 | | Incubation with the ready‐to‐use secondary buffered solution (k8002, EnVision FLEX /HRP, Agilent) for 20 minutes | | | | | | |
| Step 7 | | Sections were reacted with 3.30‐diaminobenzidine tetrahydrochloride (DAB) solution for 10 minutes | | | | | | |
| Step 8 | | Counterstain with Hematoxylin (Link) (k8008, Agilent) for 5 minutes | | | | | | |

**Supplementary Table 5a** Distribution of age and extent among subtypes, calculated and classified in the manner of 2011 St. Gallen recommendations

|  | Luminal A | | Luminal B  HER2- negative | | Luminal B  HER2-positive | | HER2  enriched | | TPN | |
| --- | --- | --- | --- | --- | --- | --- | --- | --- | --- | --- |
|  | Age (yrs) | Extent (mm) | Age (yrs) | Extent (mm) | Age  (yrs) | Extent (mm) | Age  (yrs) | Extent (mm) | Age  (yrs) | Extent (mm) |
| Minimum | 36 | 4 | 35 | 3 | 33 | 0.7 | 34 | 3.5 | 38 | 5 |
| Maximum | 87 | 80 | 74 | 120 | 76 | 115 | 83 | 150 | 88 | 80 |
| Median | 57 | 18 | 53 | 20 | 55 | 30 | 55 | 30 | 61 | 30 |
| Mean | 57 | 23.8 | 55 | 27.2 | 55 | 34 | 57 | 40.6 | 63 | 36.3 |
| **Supplementary Table 5b** Distribution of age and extent among subtypes, calculated and classified in the manner of 2013 St. Gallen recommendations | | | | | | | | | | |
|  | Luminal A | | Luminal B  HER2- negative | | Luminal B  HER2-positive | | HER2  enriched | | TPN | |
|  | Age (yrs) | Extent (mm) | Age (yrs) | Extent (mm) | Age  (yrs) | Extent (mm) | Age  (yrs) | Extent (mm) | Age (yrs) | Extent (mm) |
| Minimum | 35 | 4 | 38 | 3 | 33 | 0.7 | 34 | 3.5 | 38 | 5 |
| Maximum | 87 | 120 | 75 | 120 | 76 | 115 | 83 | 150 | 88 | 80 |
| Median | 55 | 18 | 57 | 22 | 55 | 30 | 55 | 30 | 61 | 30 |
| Mean | 56 | 24.2 | 57 | 27.2 | 55 | 34 | 57 | 40.6 | 63 | 36.3 |

**Supplementary Flowchart 1a** Distribution of subtypes in line with 2011 St. Gallen recommendations


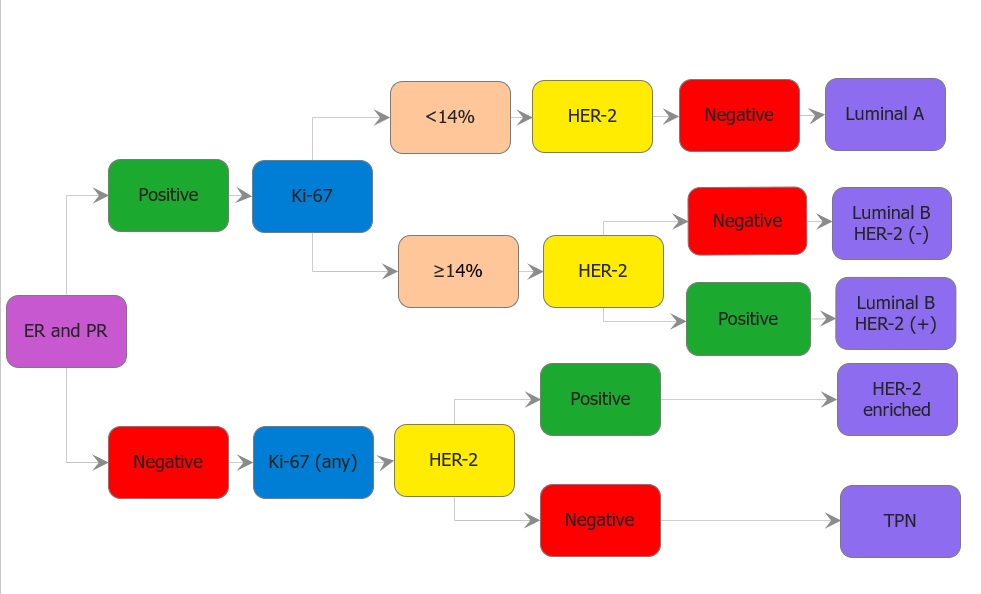


**Supplementary Flowchart 1b** Distribution of subtypes in line with 2013 St. Gallen recommendations


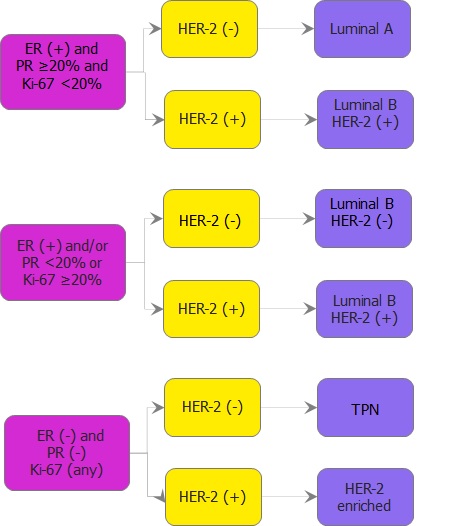

Supplement: Supplementary file 1 — Supplementary file1 (DOCX 154 KB) [file 10549_2023_7016_MOESM1_ESM.docx]
